# Supplementary material for: Cost-Effective Mapping of Genetic Interactions in Mammalian Cells
Source: Front Genet. 2021 Aug 5;12:703738. doi: 10.3389/fgene.2021.703738 (PMC8381747; doi:10.3389/fgene.2021.703738)
Supplement: Supplementary file 1 [file Data_Sheet_1.pdf]

Cost-effective mapping of genetic interactions in mammalian cells: Supplemental Figures

Arshad H. Khan[1], Desmond J. Smith[1,2]
[1] **Department of Molecular and Medical Pharmacology, David Geffen School of Medicine, UCLA, Box 951735, Los Angeles, CA 90095-1735, USA**
[2] **Corresponding author: DSmith@mednet.ucla.edu**

Figures

S1 . . . s2
S2 . . . s3
S3 . . . s3
S4 . . . s4
S5 . . . s5
S6 . . . s5
S7 . . . s6
S8 . . . s7
S9 . . . s8
S10 . . . s9
S11 . . . s10
S12 . . . s11

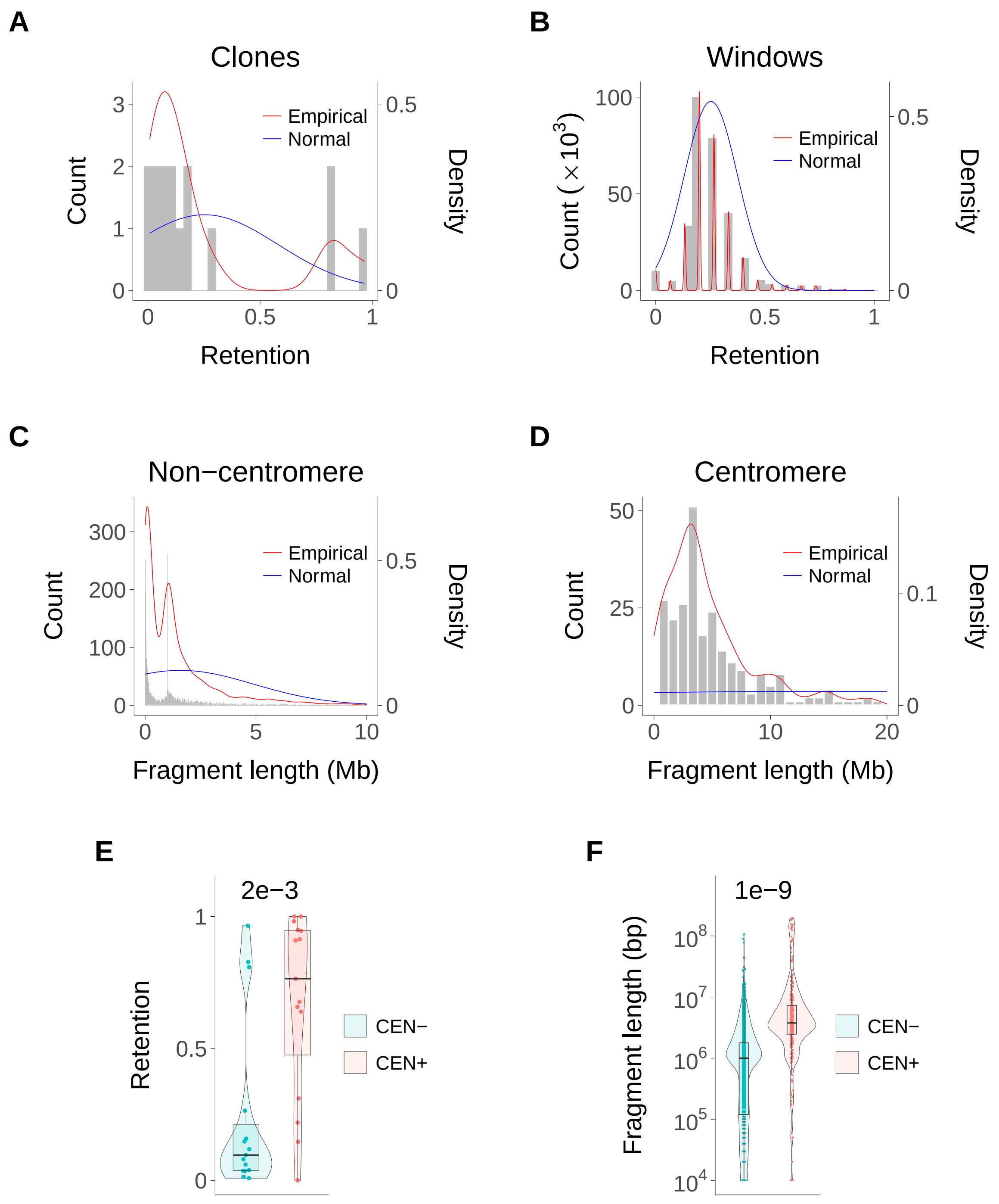

↑**Supplemental Figure S1.** Human DNA in the RH clones. (**A**) Retention for each clone. (**B**) Retention for each 1 Mb window. (**C**) Non-centromeric fragment lengths. (**D**) Centromeric fragment lengths. (**E**) Retention of centromeric DNA (CEN+) is significantly higher than non-centromeric DNA (CEN−) (0.67 ± 0.09 vs 0.24 ± 0.09, $P = 1.6 \times 10^{-3}$). (**F**) Centromeric fragment lengths are significantly longer than non-centromeric (13.5 ± 1.9 Mb vs 1.6 ± 0.05 Mb, $P = 1.4 \times 10^{-9}$).

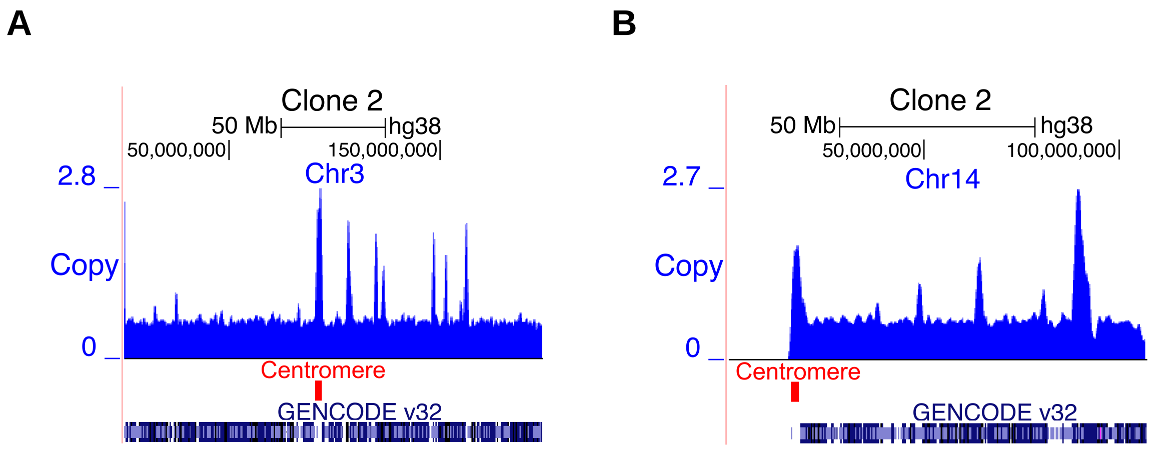

↑ **Supplemental Figure S2.** Human DNA retention in clone 2. (**A**) Human DNA copy number, clone 2, Chromosome 3. A complete human Chromosome 3 is present, in addition to smaller fragments. (**B**) Clone 2, Chromosome 14. A complete human Chromosome 14 is present, in addition to smaller fragments.

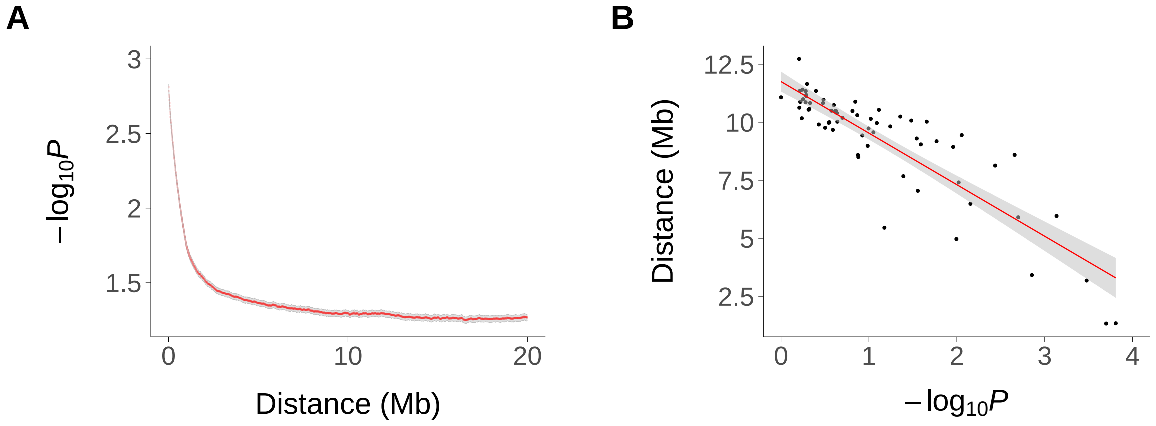

↑ **Supplemental Figure S3.** *Cis* linkage disequilibrium for RH-Seq clones. (**A**) Significance values for *cis* linked windows ($-\log_{10} P$) plotted against distance. (**B**) Distance of *cis* linked windows plotted against $-\log_{10} P$. Gray, 95% confidence intervals.

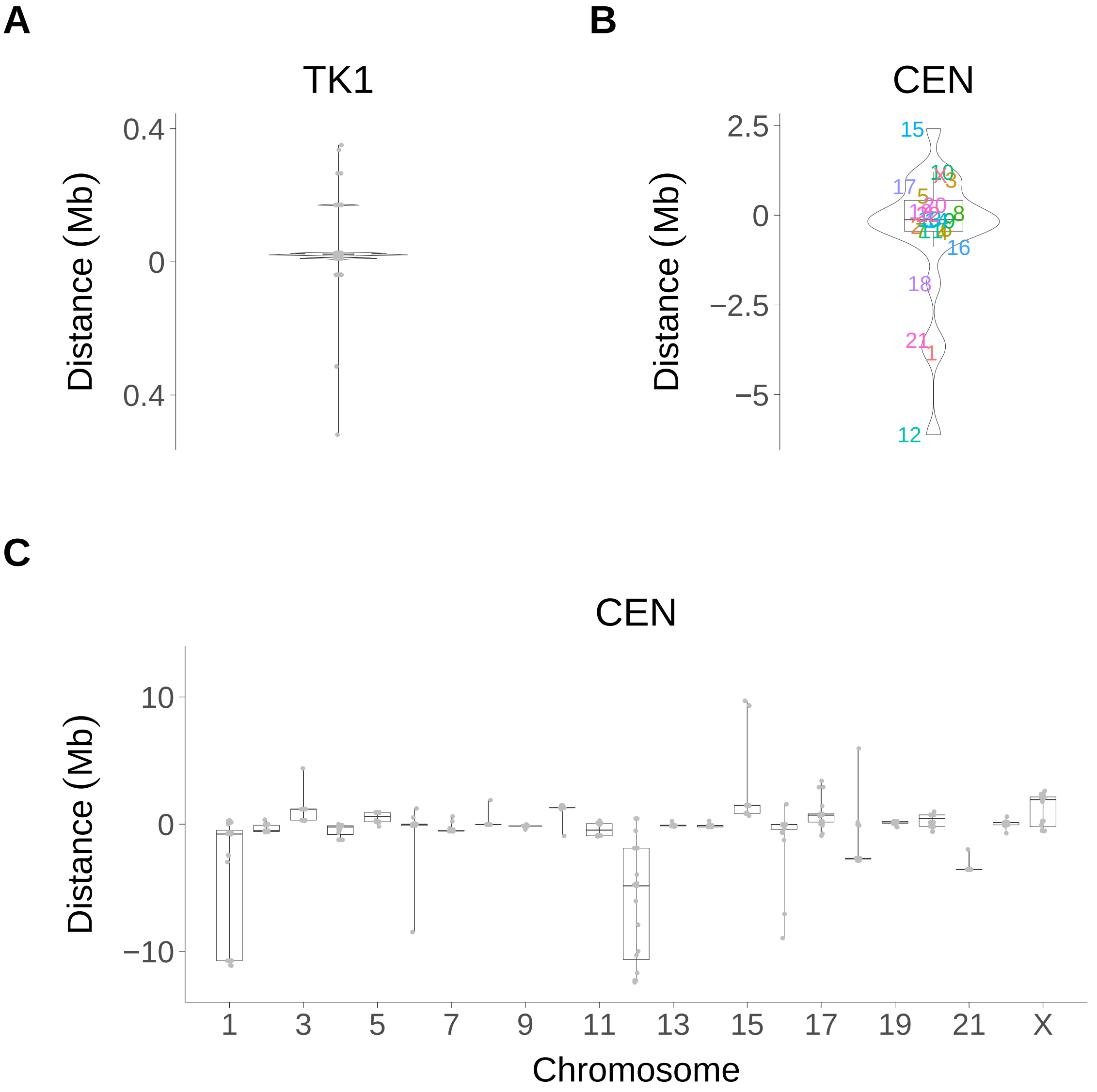

↥ **Supplemental Figure S4.** Mapping accuracy. (**A**) TK1. (**B**) Centromeres averaged across clones. Colored labels indicate chromosomes. (**C**) Centromeres vs chromosome. Bootstrapping used to evaluate variance.

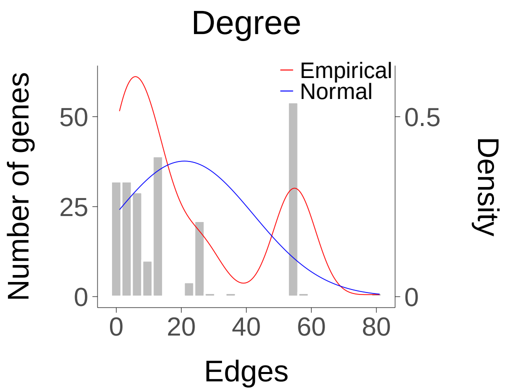

↑ **Supplemental Figure S5.** Degree of RH-Seq network.

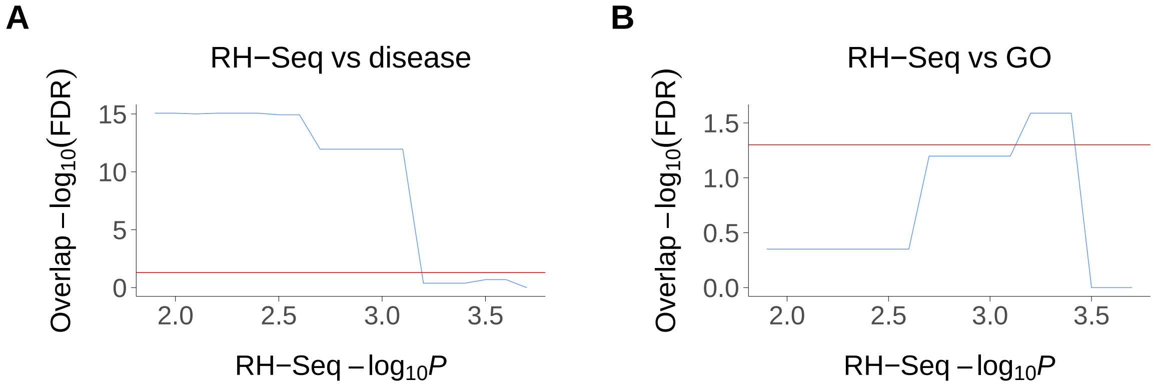

↑ **Supplemental Figure S6.** Overlaps between the RH-Seq network and gene-disease and gene ontology (GO) networks. (**A**) Significant overlap of RH-Seq network and gene-disease network, thresholded on RH-Seq $-\log_{10} P$. (**B**) RH-Seq network and GO network. Horizontal red lines, FDR $= 0.05$.

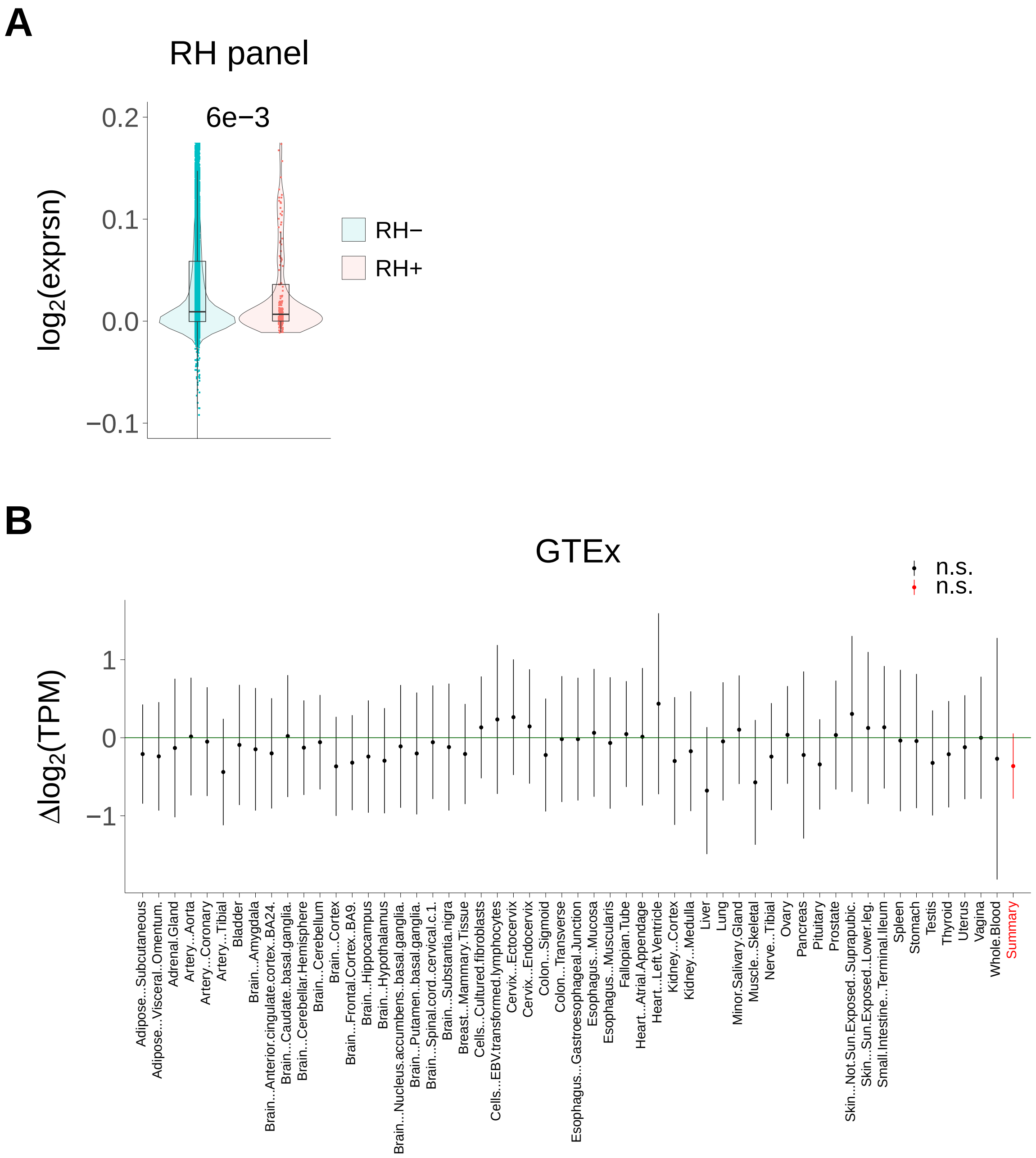

↑ **Supplemental Figure S7.** RH-Seq network and gene expression. (**A**) Genes in the RH-Seq network (RH+) show significantly lower expression than genes outside the network (RH−) using microarray data from a human RH panel ($P = 5.7 \times 10^{-3}$). (**B**) No significant differences in expression for RH-Seq network genes compared to non-network genes in various tissues using GTEx. $\Delta\log_2(\text{TPM}) < 0$ means decreased expression for genes in RH-Seq network compared to non-network genes. Transcripts per million (TPM) thresholded at ≥ 5.

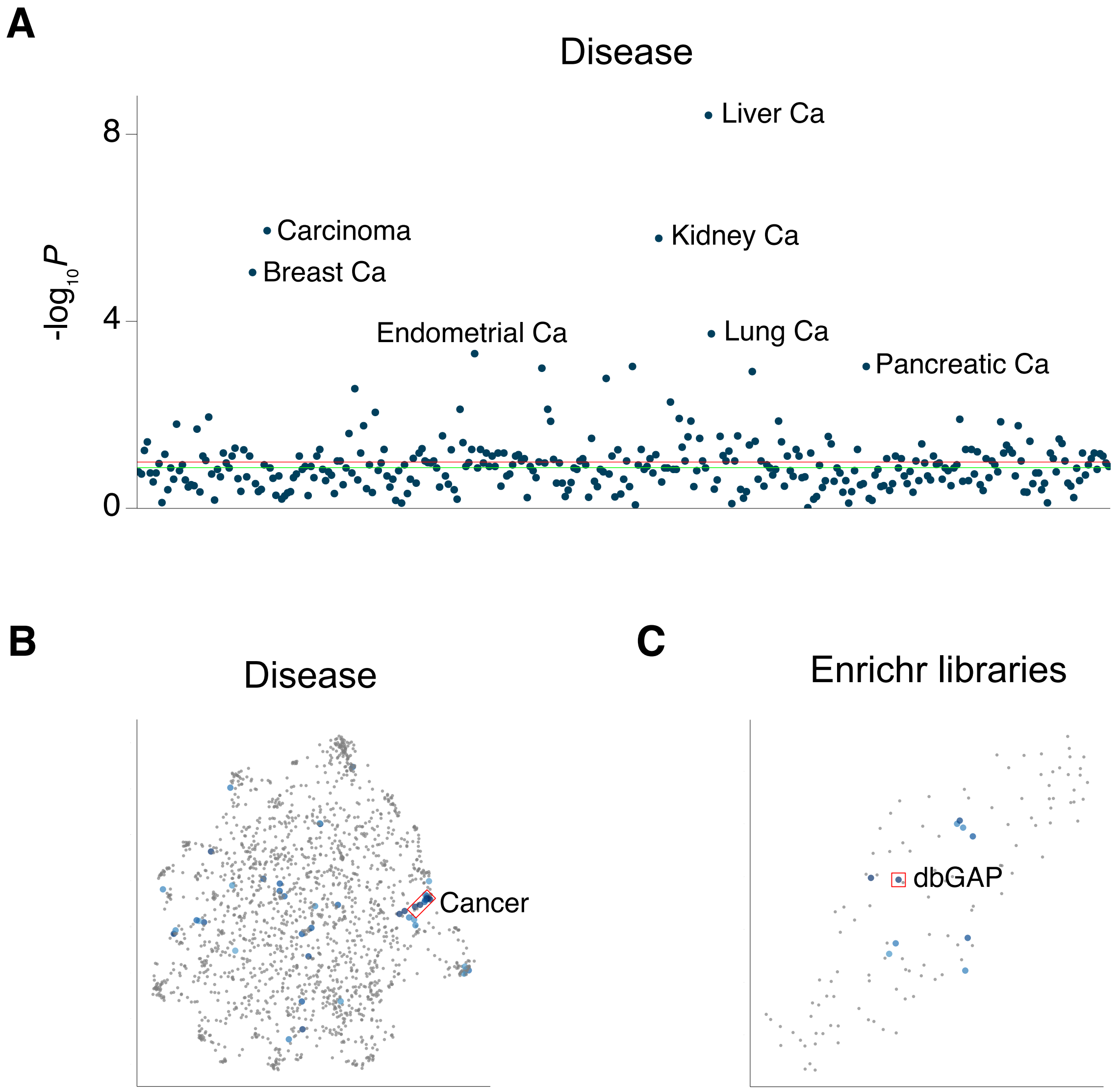

↑ **Supplemental Figure S8.** Enrichr analysis of genes in RH-Seq network. (**A**) Manhattan plot of human disease categories shows prominence of cancer. Green horizontal line, $P = 0.05$, red horizontal line, FDR $= 0.05$. (**B**) Scatterplot of human disease categories, showing significant over-representation of cancer. Related categories cluster together. Coordinates, arbitrary units. (**C**) Enrichr libraries with increased representation of human interaction genes, showing significant over-representation in dbGaP.

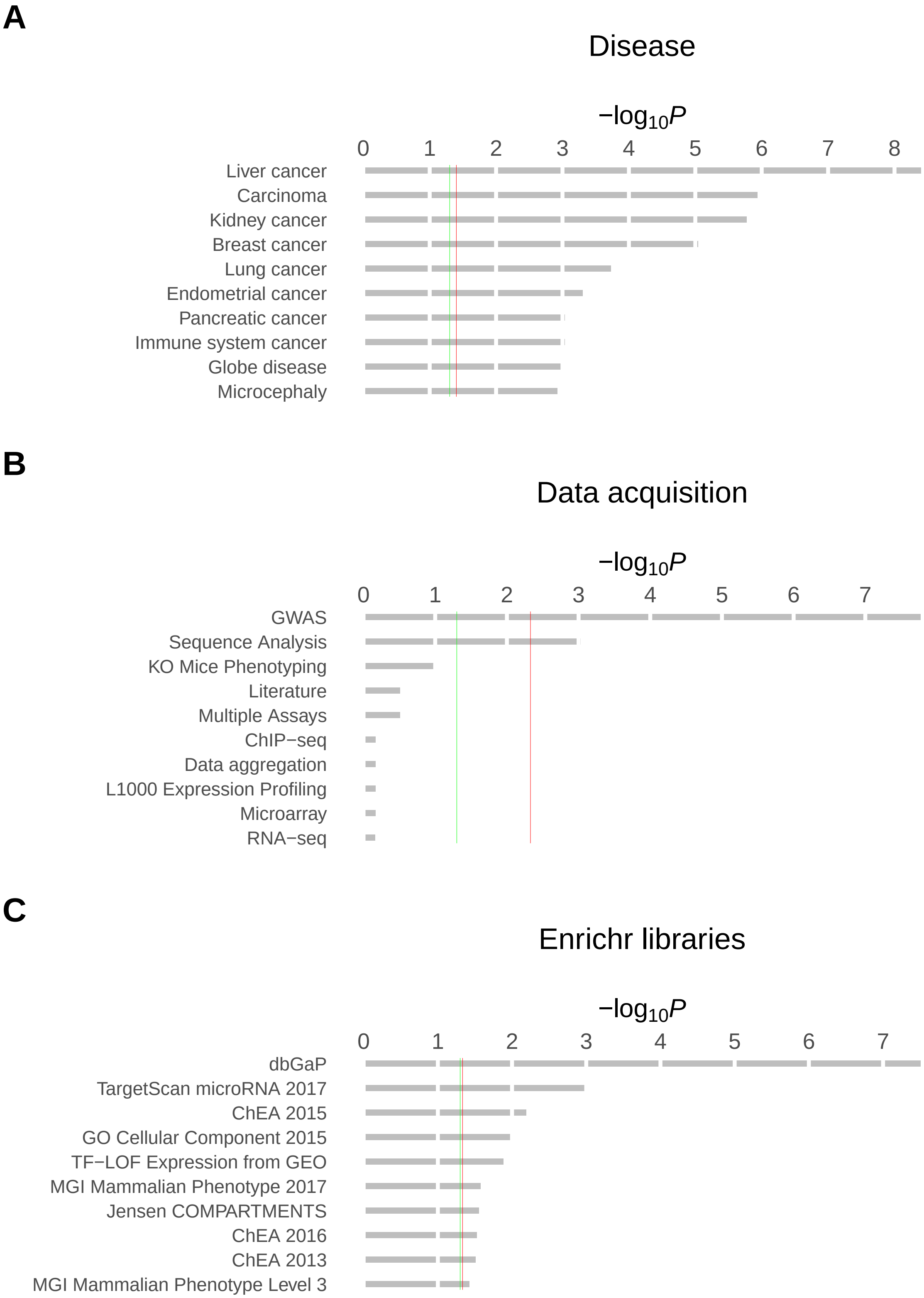

↑ **Supplemental Figure S9.** Enrichr analysis of genes in RH-Seq network, showing $-\log_{10} P$ values. (**A**) Disease categories, revealing prominence of cancer. (**B**) Most popular data acquisition method, with GWAS most significant. (**C**) Enrichr libraries, with dbGAP most significant. Green vertical lines, $P = 0.05$, red vertical lines, FDR $= 0.05$.

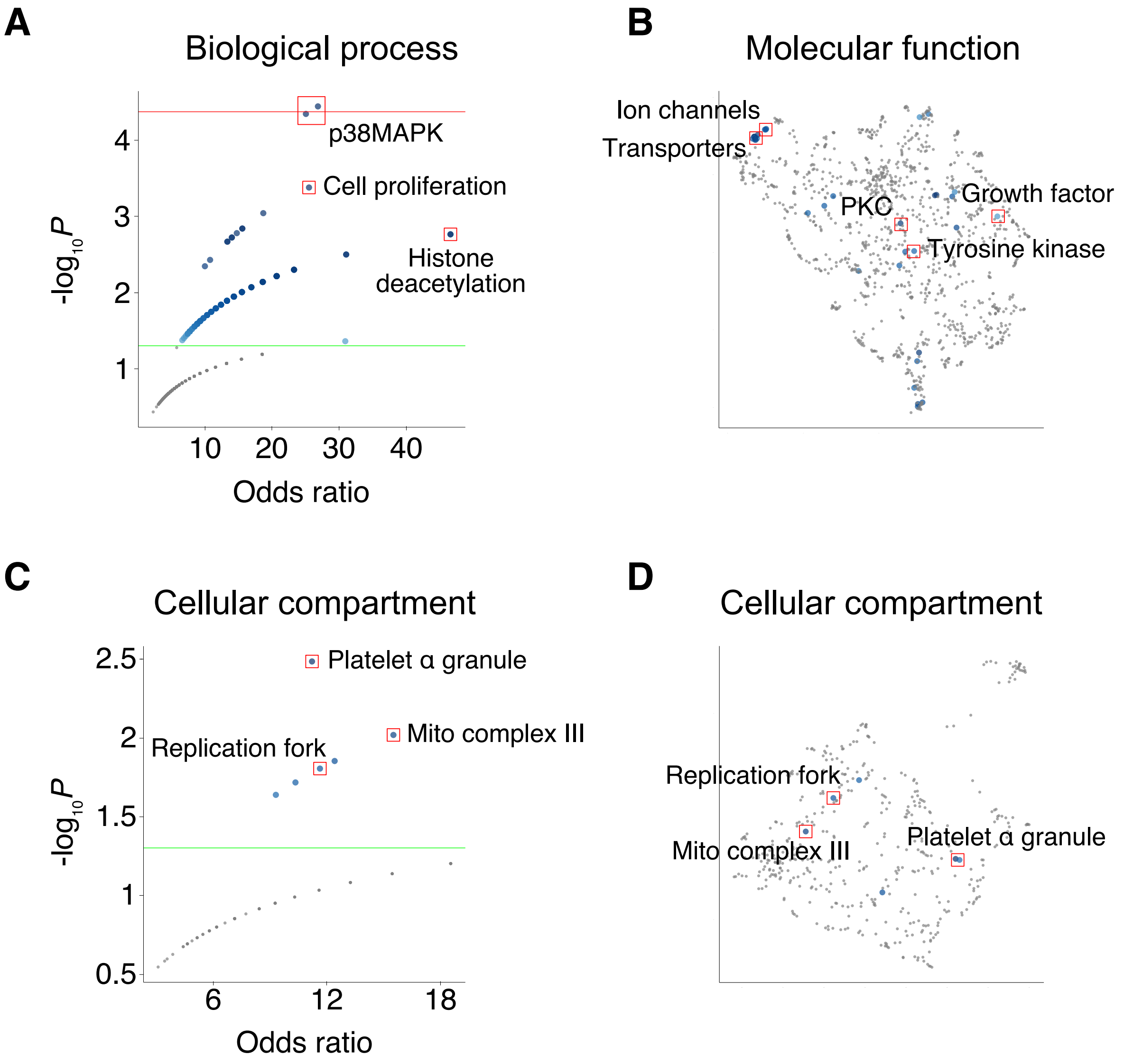

↑ **Supplemental Figure S10.** GO analysis of human genetic interactions using Enrichr. (**A**) Volcano plot of GO biological process. (**B**) Scatterplot of GO molecular function. Related categories cluster together. Coordinates, arbitrary units. (**C**) Volcano plot of GO cellular compartment. (**D**) Scatterplot of GO cellular compartment. Categories related to cell proliferation are enriched. Green horizontal lines, $P = 0.05$, red horizontal line, FDR $= 0.05$.

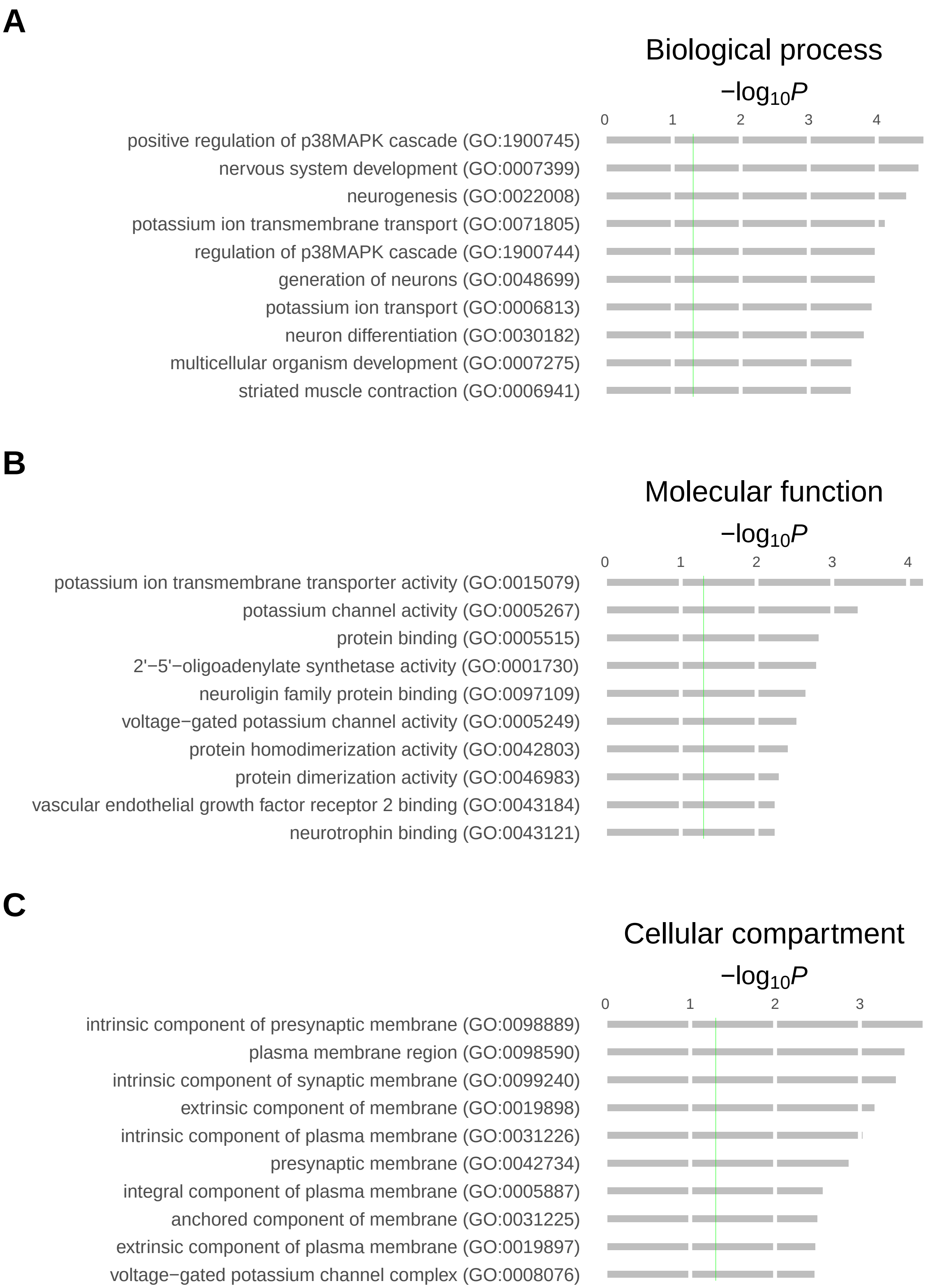

↑ **Supplemental Figure S11.** GO analysis of human genetic interactions using PANTHER Overrepresentation Test. (**A**) Biological process. (**B**) Molecular function. (**C**) Cellular compartment. Vertical green lines, $P = 0.05$.

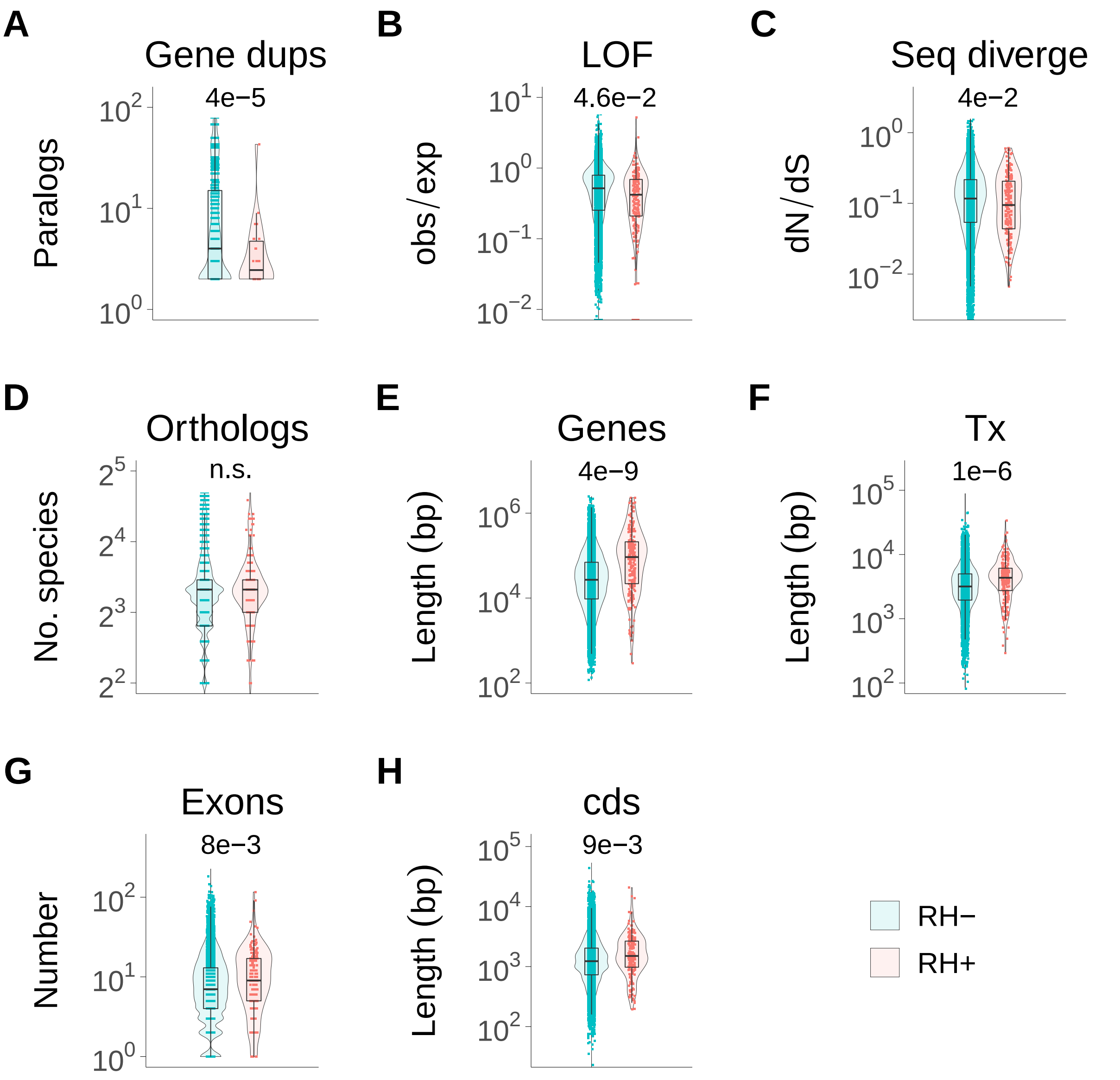

↑ **Supplemental Figure S12.** Evolutionary properties of RH-Seq network genes. (**A**) RH-Seq network genes show decreased gene duplications. (**B**) Decreased tolerance to loss-of-function (LOF) variants. (**C**) Decreased mouse-human sequence divergence. (**D**) No significant difference in the number of orthologs. (**E**) RH-Seq network genes show increased gene lengths. (**F**) Increased transcript lengths. (**G**) Increased exon numbers. (**H**) Increased coding sequence (cds) lengths. RH+, genes in RH-Seq network; RH−, genes outside network. *P* values shown above plots.
